# Hydroclimatic record from an Altiplano cushion peatland (24°S) indicates large-scale reorganisation of atmospheric circulation for the late Holocene

**DOI:** 10.1371/journal.pone.0277027

**Published:** 2022-11-10

**Authors:** Andreas Lücke, Sebastian Kock, Holger Wissel, Julio J. Kulemeyer, Liliana C. Lupo, Frank Schäbitz, Karsten Schittek

**Affiliations:** 1 Agrosphere Institute IBG-3, Institute of Bio- and Geosciences, Forschungszentrum Jülich GmbH, Jülich, Germany; 2 Institute of Geography Education, University of Cologne, Cologne, Germany; 3 Facultad de Ingeniería/Agrarias, Instituto de Ecorregiones Andinas (INECOA-CONICET), National University of Jujuy, Jujuy, Argentina; 4 Laboratorio de Palinología, Facultad de Ciencias Agrarias, Instituto de Ecorregiones Andinas (INECOA-CONICET), National University of Jujuy, Jujuy, Argentina; Woods Hole Oceanographic Institution, UNITED STATES

## Abstract

The hydroclimate of South America is characterized by the South American summer monsoon (SASM), a tropical atmospheric circulation that induces a summer precipitation regime, and the Southern Hemisphere Westerlies (SHW), an extratropical atmospheric circulation that induces a winter precipitation regime. Stretched between these two systems is a NW-SE-oriented region dominated by descending air masses, resulting in the South American subtropical dry zone (SASDZ), also known as the arid diagonal. We investigated the Cerro Tuzgle cushion peatland (CTP) located on the Argentine Altiplano, north of the present-day SASDZ. Previous work revealed that the CTP was consistently in the SASM regime during the last 2900 cal yr BP. Here, we extend the CTP record to the middle Holocene covering the last 7200 cal yr BP to gain further knowledge of the Holocene development of the SASM and potential modulations of the SASDZ. The prominent feature of the entire record is a distinct and lasting transition centred around 3100 cal yr BP characterized by declining minerogenic content, increasing organic carbon content, rising stable carbon isotope values of organic matter and cellulose, and increasing stable oxygen isotope values of cellulose. We interpret this specific proxy pattern as a hydroclimatic transition towards less arid conditions at the CTP after 3100 cal yr BP. The transition corresponds with the end of the continuous Holocene strengthening of the SASM between 3500 cal yr BP and 3000 cal yr BP indicated by proxy records from north and east of the CTP. The CTP does not reflect this strengthening of the SASM and rather exhibits a threshold response indicating the effective establishment of the SASM summer precipitation regime at 24°S. This suggests that moisture supply during a more arid middle Holocene was provided by isotopically depleted precipitation, while moisture supply after the transition originated from isotopically enriched SASM summer precipitation. Concurrent hydroclimatic changes in the SHW winter precipitation regime south of the SASDZ are documented in a distinct lake level rise of Laguna Aculeo (33°50´S) around 3200 cal yr BP. These coinciding hydrological changes of the SASM and the SHW precipitation regimes indicate larger scale reorganisations of atmospheric circulation components, potentially connected to major modulations of the SASDZ. Thus, our CTP record sheds light on the middle to late Holocene development of the SASM at its southern limit and corroborates connections between the tropical and extratropical hydroclimate of South America.

## Introduction

Two major atmospheric circulation systems determine the climate and precipitation regimes of the South American continent. Hydroclimate in tropical regions is governed by the SASM with a summer precipitation regime, while extratropical regions are governed by the SHW with a winter precipitation regime [1]. Very low precipitation amounts are typical for a region between these circulation systems crossing the complete continent from NW to SE, known as the arid diagonal. This arid zone develops dynamically as a result of the confluence of descending, dry air masses from the southern branch of the South American Hadley cell and the northern branch of the Ferrel cell. Thus, the arid diagonal is the South American subtropical dry zone (SASDZ), effectively separating the tropics and the extratropics [2, 3].

Extended knowledge of Holocene changes in precipitation regimes on both sides of the SASDZ is of widespread interest with respect to future developments of tropical and extratropical circulation systems. Climate models project a southward shift of the intertropical convergence zone (ITCZ) for the eastern Pacific and Atlantic oceans and indicate large-scale hydrological changes in South America in the future [4]. However, simulations of the twentieth and twenty-first centuries project a significantly smaller zonally averaged poleward extension of the tropical circulation and the subtropical dry zone than already observed in recent decades [5]. Southern Hemisphere semi-arid regions have recently experienced a reduction of rainfall in austral autumn [2, 6]. While the rainfall decline in southeastern Australia can be explained by a poleward shift of the subtropical dry zone, similar evidence for a poleward shift in the southern Chilean sector is missing despite the occurrence of autumn drying [2]. This regionally non-uniform behaviour raises questions with respect to the connection between meridional changes in precipitation and changes in atmospheric circulation such as Hadley cell shifts.

Knowledge about the southern limit of the SASM or the position of the SASDZ during the Holocene can thus provide crucial information to evaluate future model projections under different climate change scenarios. Changes in hydrology and rainfall regimes have been reported for various time slices and different regions of South America during the Holocene [7–9]. Due to the limited availability of suitable archives and differences in the quality and explanatory power of different proxy variables, evidence is often inconsistent. For the Atacama Desert and the southern Central Andes, abiotic proxy data and paleosols indicate an extremely dry middle Holocene, whereas plant macrofossils in rodent middens indicate more humid conditions than today [10, 11]. This discrepancy underlines the need for more and spatially distributed proxy archives for a comprehensive understanding of past South American climate variations and their forcing mechanisms.

Dynamic connections between tropical and extratropical precipitation, between the SASM and the SHW as well as with the South Pacific Subtropical Anticyclone, have been suggested as an explanation for Holocene precipitation changes in South America. Such a connection between precipitation in Chile and the intensity and position of the South Pacific anticyclone is highlighted by modern observations [12]. Accordingly, Markgraf [13] explained an inverse relation between cooler and wetter intervals in northern Patagonia and drier intervals in central Chile observed in the past with shifts in the Pacific subtropical anticyclone. As the subtropical anticyclone determined the location of the westerly storm tracks, a further northward position than average reduced winter rains in central Chile whereas middle and high-latitude regions received increased precipitation [13]. Variations in reconstructed precipitation during the last 7000 cal yr BP in southern Chile were related to variations in the latitudinal position and intensity changes of the SHW [14, 15]. This suggested a close connection of rainfall variability in Chile to sea surface temperatures in the eastern South Pacific and a simultaneous change in the tropical climate system [14, 15]. Similarly, several alterations between more humid and more arid phases during the last 10,000 cal yr BP were indicated by pollen analysis from a swamp forest on the semi-arid coast of Chile (32°) at the northern influence zone of the SHW [16]. This pattern was explained by variations in the extent of the SHW caused either by latitudinal displacements from the mean position of the SHW belt or by changes in the intensity of the South Pacific subtropical anticyclone [16].

Modern observations also show widespread teleconnections between tropical and extratropical circulation in South America with the meridional Hadley circulation, the zonal Walker circulation and the El Niño–Southern Oscillation (ENSO) system in the larger South Pacific region [17]. An expanded Hadley circulation in the Southern Hemisphere is associated with a poleward expansion of the tropical wet zone in the Asia-Pacific sector and with La Niña-like conditions [18]. Because of the relation between the extent of the Hadley cell and ENSO, La Niña-like conditions tend to contract the South American Hadley cell during austral spring and summer [3, 18]. In the southern Central Andes and the Altiplano, ENSO-related atmospheric circulation anomalies show a tendency for wet conditions during La Niña years [1]. Moreover, the strength and position of the SHW storm tracks is affected by heating (cooling) over the South Pacific convergence zone in the Australian region through influences on the strength of the austral summer South Pacific subtropical anticyclone off South America [19, 20].

In recent years, high-elevation cushion peatlands from the semi-arid regions of the Andes have become valuable archives of South American past climates and SASM variability [21–26]. The response of these vascular plant dominated peatlands to hydroclimatic changes in the semi-arid, moisture-limited environment of the Altiplano–Puna plateau is visible in plant growth and primary production and the stable isotope composition of the accumulated peat. Modern field studies have shown that stable oxygen isotope values (δ^18^O) of vascular plant organic matter offer a fairly good reflection of the isotopic composition of precipitation and peat water [e.g. 22, 27] and, thus, can be used to infer past variations of the amount and origin of precipitation from respective peat cores.

The Cerro Tuzgle cushion peatland (CTP) is one of the few sites in this region to improve our lack of knowledge on the longer-term evolution of the SASM on the southern Altiplano-Puna plateau during the Holocene. Here, we extend the CTP record [22, 25] to the middle Holocene covering the last 7200 cal yr BP. Our aim was to investigate the timing and causes of Holocene hydroclimatic changes on the southern Altiplano at the southern edge of the SASM realm (tropical precipitation), north of the present-day SASDZ (arid diagonal). In our study, we applied measures of vegetation composition and primary production, the contribution of clastic material, as well as of moisture availability and hydroclimate, such as total organic carbon content, organic carbon isotopes and cellulose oxygen isotopes. We present evidence for a prominent hydroclimatic transition centred at 3100 cal years BP. We discuss this transition in relation to the longer-term history of the SASM and within the context of hydroclimatic connections between tropical and extratropical precipitation across the SASDZ. We also show relations with hydroclimatic archives in the larger South Pacific region, indicating relations to the dynamics of the South American Hadley cell and the Walker circulation.

## Study area

**Fig 1:**
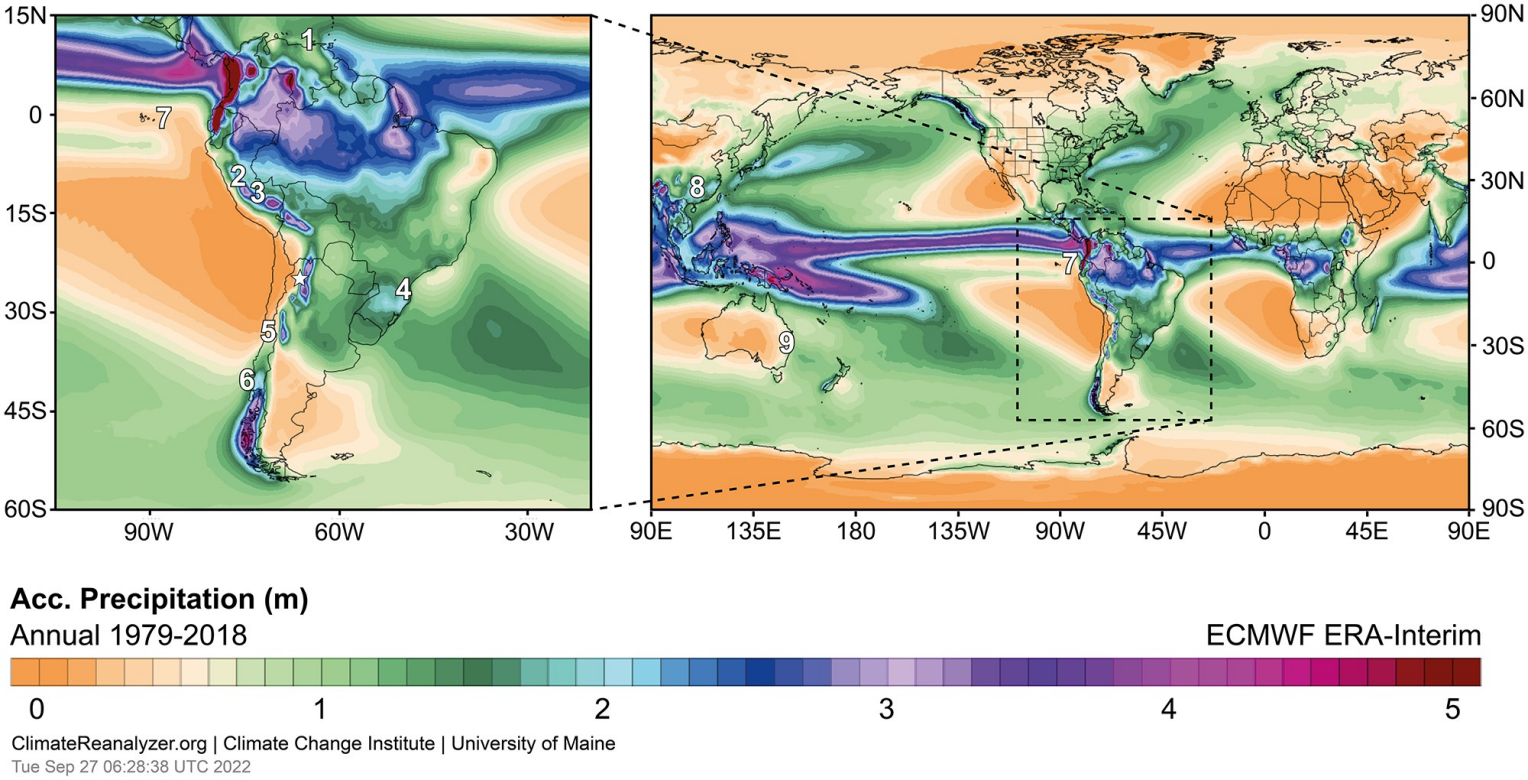
**Left: Mean annual precipitation (1979–2018) in South America with locations of the study site CTP (✰) and of other sites mentioned in the text: 1** Cariaco Basin (10°42′N, 65°10′W), **2** Laguna Pumacocha (10.70°S, 76.06°W), **3** Huagapo cave (11.27°S, 75.79°W), **4** Botuvera cave (27°13′S, 49°09′W), **5** Laguna Acuelo (33°50’S, 70°54’W), **6** GeoB 3313–1 (41°S, 74°W). The SASDZ or arid diagonal is visible in the region of precipitation minima represented by orange colours. **Right: Same as before but for the South Pacific realm with additional sites mentioned in the text: 7** El Junco lake, Galápagos (0.8°S, 89.3°W), **8** Dongge cave, East Asia (25°17’N, 108°5’E), **9** Swallow Lagoon, Australia (27°29′S, 153°27′E). Maps from Climate Reanalyzer (https://ClimateReanalyzer.org), Climate Change Institute, University of Maine, USA.

The CTP (24°09’ S, 66°24’ W) is located in the northwest Argentine Andes, Jujuy province, close to the Cerro Tuzgle volcano at 4,350 m a.s.l. (Fig 1). This region is part of the internally drained Puna, comprising the southern part of the Altiplano-Puna plateau [28, 29] where the CTP is situated in the eastern, semi-humid part of the Argentine Puna [30, 31]. The study site is located north of the SASDZ, or the arid diagonal, the frontier area between the tropical and extratropical precipitation regimes [1, 32]. Under present-day conditions, precipitation predominantly reaches the CTP from northwesterly directions and, therefore, with the SASM. The nearest weather station at the Concordia mine (4,144 m a.s.l., available data: 1950–1990) shows that more than 90% of mean annual rainfall (109 mm) occurs between November and March [22]. Cold frontal or cut-off events that lead to precipitation in austral winter are rare. Annual temperatures range between 2°C and 10°C with mean annual temperatures of 6.9°C during the observation period.

The CTP is situated in the valley of an ephemeral stream between comparatively flat slopes consisting of Ordovician volcanic rocks and Cretaceous conglomerates and sandstones, and is protected against increased allochthonous sediment input from lateral stream channels or valleys during extreme rainfall events [25]. A currently inactive alluvial fan in the southernmost section of the peatland developed a natural dam and led to the continuous accumulation of peat in the past. Modern vegetation in the peatland is predominantly characterized by *Oxychloe andina* (*O*. *andina*) supplemented by *Zameioscirpus muticus* (*Z*. *muticus)*. *O*. *andina* grows in dense cushions with leaves at their tops and rhizomes at the bottom. The leaves are lignified and have a prickle at their tops, potentially as protection against grazing [25]. In contrast, *Z*. *muticus* grows in small gaps and fissures of the *O*. *andina* cushions. *Z*. *muticus* has a higher physiological and ecological tolerance, showing greater resistance against drought and disturbances, for instance. However, the species cannot compete with the growth of *O*. *andina* under wet conditions [33]. Cushion peatlands are comparably less vulnerable to water availability due to their cushion-like growth increasing their resistance to water fluctuations [34]. Discharge for the ephemeral stream and for the CTP originates from springs and from rainfall events with run-off from the surrounding slopes. The surface of the CTP is characterized by microtopographical heterogeneity where the cushions are interspersed with shallow pools of different sizes that can be connected but which also occur isolated without a superficial connection. The degree of the evaporative enrichment of stable isotopes in the surface water of those pools is determined by relative air humidity, air temperature and the water residence time, i.e. the evaporation to inflow ratio of the reservoir [35]. At present, oxygen isotope enrichment in pools on the CTP vary between 1.0‰ and 5.0‰ but can exceed 10.0‰ depending on pool size and hydrologic connectivity [22]. However, a peat water sample extracted and sampled in November 2014 revealed an enrichment that was only 1.5‰ above the expected moisture source [22].

## Material and methods

### Coring, sub-sampling and geochemical measurements

The core Tuz-694 was retrieved by percussion coring in December 2012 in 1 m segments, reaching a total depth of 8 m. Cores were stored in a cool environment until opening at the University of Cologne. The cores were opened lengthwise in the laboratory, cut into two halves with a modified core saw, photographed, and described lithologically. One core half was used for major and trace element scanning analyses at GEOPOLAR, University of Bremen. This core half was cut in 1 cm slices from which the contact surfaces with the core liner were removed. These samples were used for cellulose extraction and stable carbon and oxygen isotope analyses on cellulose. The second core half was prepared accordingly for element content analyses, stable organic carbon and total nitrogen isotope analyses on bulk material (Institute of Bio- and Geosciences–Agrosphere, Forschungszentrum Jülich GmbH) and further prepared for microfossil/macrofossil analyses (University of Cologne).

On the first core half, major and trace elements were measured with a resolution of 2 mm on an ITRAX X-ray fluorescence (XRF) core scanner (CS-8, Cox Analytical Systems, SE) (GEOPOLAR, University of Bremen). Measurements were performed with a molybdenum (Mo) tube at 30 kV and 10 mA for an exposure time of 10 s [36]. Results are reported as total counts (cnts) for the measured elements Ti, K, S, Ca, Mn and Fe selected for interpretation.

### Composite profile and radiocarbon dating

The upper sections of the second and third core segments were determined to be caving material and thus discarded [22]. The retrieved core segments were compacted unequally during the coring due to the high water content. To build a composite profile, the core segments were linearly decompacted by a correction factor (core length/compacted length). Decompacted core segments were used to build the composite profile that serves as a basis for the age-depth model and for all core depths mentioned in the text.

Twenty-seven bulk peat samples were sent to Poznań Radiocarbon Laboratory, Poland, for AMS radiocarbon dating. The ^14^C ages (Table 1) were calibrated against the SHCal20 curve [37] with CALIB 8.2 [38]. All AMS ^14^C ages were used to establish the chronostratigraphy for the CTP composite profile. The peat surface (CE 2012) was used as an additional tie point. The age-depth model was calculated with the Bacon age-modelling software (package ‘rbacon’, version 2.5.3 [39]) in R [40]. The Bacon software was used with the default settings, with the exception that the section thickness was set to 5. This set-up achieved good runs with stationary performance of iterations and reduced confidence ranges (mean 95% = ~263 yr). All ages are reported as calibrated years before 1950 (cal yr BP).

**Table 1:** AMS radiocarbon dates from the CTP calibrated with CALIB 8.2 [38] and the Southern Hemisphere calibration curve (SHCal20, [37]). Modelled ages were obtained with age-depth modelling software Bacon [39].

Lab-ID	Composite depth (cm)	Radiocarbon age (^14^C BP)	Radiocarbon error (± 1σ)	Calibrated age (cal yr BP)	Minimum age 2σ (cal yr BP)	Maximum age 2σ (cal yr BP)	Modelled age (cal yr BP)	Minimum age* (cal yr BP)	Maximum age* (cal yr BP)
Poz-56032	38.1	600	35	554	512	632	545	440	629
Poz-56034	78.1	1095	30	950	919	1053	939	825	1025
Poz-56035	103	1245	25	1115	999	1257	1131	1065	1228
Poz-66440	143.9	1620	30	1469	1377	1535	1469	1388	1545
Poz-56036	160.2	1715	30	1575	1520	1698	1602	1530	1695
Poz-66442	186.7	1960	30	1864	1749	1989	1889	1792	1994
Poz-56037	233.6	2475	35	2495	2353	2704	2585	2391	2703
Poz-56038	245.5	2705	30	2778	2740	2851	2759	2591	2839
Poz-56039	257.5	2820	30	2882	2778	2992	2864	2787	2944
Poz-56040	281.4	2965	30	3078	2960	3207	3046	2958	3142
Poz-56041	306.8	3160	35	3335	3217	3442	3217	3110	3299
Poz-56042	328.4	3135	35	3297	3180	3392	3323	3239	3406
Poz-56051	347.3	3150	35	3318	3211	3439	3422	3340	3554
Poz-56152	393.2	3600	40	3861	3699	3981	3869	3742	3981
Poz-56153	403.7	3635	35	3907	3728	4079	3980	3862	4109
Poz-56154	441.0	4035	35	4475	4298	4610	4554	4400	4795
Poz-56155	464.9	4500	35	5129	4886	5297	5116	4903	5284
Poz-56157	490.3	5060	40	5775	5608	5900	5721	5599	5869
Poz-57068	501.1	5235	30	5962	5898	6170	5887	5744	5979
Poz-118426	507.8	5260	40	5984	5905	6178	5943	5784	6036
Poz-57069	587.8	5850	35	6618	6497	6733	6507	6380	6621
Poz-118298	639.2	5950	40	6736	6637	6882	6771	6674	6891
Poz-118299	668.9	5970	40	6761	6662	6885	6939	6829	7114
Poz-57072	693.2	6220	35	7076	6953	7240	7139	7019	7257
Poz-56159	707.1	7040	50	7835	7699	7937	7287	7148	7455
Poz-56161	743.9	6810	50	7625	7513	7707	7586	7449	7692
Poz-56162	778.6	6880	40	7674	7587	7779	7816	7674	7961

* 95% confidence interval

### Plant macrofossils

For plant macrofossil analyses, subsamples at 8 cm intervals were selected. In the middle section of the core, where the peat matrix is interrupted by layers of inorganic debris more repeatedly, the intervals were more closely spaced. Samples of 5 cm³ were treated and sieved according to the techniques described in [25]. Plant tissues (mostly rhizome and root remains) were determined in the >250 μm sieve fraction under a dissecting microscope. For identification, our own reference collection and published keys were used [41, 42]. The presence of identifiable plant remains was recorded by an abundance scale in three categories: I. *Oxychloe* >90%, Scirpeae <10%; II. *Oxychloe* >70%-90%, Scirpeae <10%-30%; III. *Oxychloe* >50%-70%, Scirpeae >30%-50%.

### Stable isotopes

For organic carbon content (TOC), total nitrogen content (TN), stable organic carbon (δ^13^C_org_) and total nitrogen isotope (δ^15^N_bulk_) analyses, bulk peat samples were freeze-dried and milled (Retsch, MM 400). Tests with 5% HCl revealed that decalcification was not necessary for organic carbon determination. Samples were weighted in tin capsules, combusted at 1080°C in an elemental analyser (EuroEA, Eurovector) and measured online with an isotope ratio mass spectrometer (Isoprime, Micromass). The analyses of δ^13^C_org_ and TOC as well as δ^15^N_bulk_ and TN were performed within a single measurement run. The calibration of laboratory standards and scale-normalisation of δ^13^C raw values was based on the International Atomic Energy Agency (IAEA) reference standards IAEA-CH6 (δ^13^C = -10.45‰) and IAEA-CH7 (δ^13^C = -32.15‰), and the United States Geological Survey (USGS) standard USGS24 (δ^13^C = -16.05‰). The calibration of laboratory standards and scale-normalisation of δ^15^N raw values was based on the international reference standards IAEA-N-2 (δ^15^N = 20.3‰), IAEA-N-1 (δ^15^N = 0.4‰) and USGS25 (δ^15^N = -30.4‰). TN and TOC contents were determined by peak integration and calibrated against certified elemental standards.

Prior to cellulose extraction, bulk peat samples were disaggregated with 0.1 M NaOH at room temperature for 16 h on a shaker and subsequently sieved into three size fractions at 200 μm and 1000 μm (<200 μm, 200–1000 μm, >1000 μm). Below a composite depth of approximately 3m, sample amounts in the largest fraction were insufficient for cellulose extraction. Only the size fraction 200–1000 μm was used for cellulose extraction below a composite depth of 3 m, as [22] have shown that the two smaller size fractions are well correlated. Cellulose was chemically extracted from the 200–1000 μm fraction samples using the CUAM (cuprammonium) protocol described in [43]. All cellulose data reported here refer to the size fraction 200–1000 μm. The C/O ratio of the extracted cellulose (mean: 0.91, std: 0.03), which is used as a quality indicator for the purity of the extracted cellulose, was always in the range expected from the stoichiometry. Due to very low organic matter content, cellulose yields were insufficient for stable isotope analyses at composite depths of 350 cm to 380 cm (3439–3736 cal yr BP), 407 cm to 417 cm (4020–4182 cal yr BP), 425 cm to 438 cm (4302–4504 cal yr BP), 495 to 499 cm (5791–5864 cal yr BP) and 530 cm to 566 cm (6100–6355 cal yr BP).

For organic stable carbon isotope analyses, about 250 μg of cellulose (δ^13^C_cell_) was weighed in tin foil capsules. Samples were combusted at 1050°C with excess oxygen in an elemental analyzer (EuroEA, Eurovector, Italy) and measured online with a coupled isotope ratio mass spectrometer (IsoPrime, GV-Instruments, UK). Carbon content (C) was determined by peak integration (*m/z* 44 and 45) and calibrated against a certified elemental standard. The International Atomic Energy Agency (IAEA) reference standards IAEA-CH6 (δ^13^C = -10.45 ‰) and IAEA-CH7 (δ^13^C = -32.15 ‰), and the United States Geological Survey (USGS) standard USGS24 (δ^13^C = -16.05 ‰) were used to calibrate laboratory isotope standards and to scale-normalise raw data. For cellulose stable oxygen isotope (δ^18^O_cell_) analyses, about 275μg of freeze-dried cellulose was weighed in silver capsules, crimped and stored for at least 24 h in a vacuum drier at 110°C before analysis. Samples were pyrolysed at 1450°C using a high-temperature pyrolysis analyzer (HT-O, HEKAtech, Germany) and measured online with a coupled IRMS (Isoprime, GV Instruments, UK). Oxygen content (O) was determined by peak integration of (m/z 28 and 29) and calibrated against a certified elemental standard. The reference standards IAEA-601 (δ^18^O = 23.14 ‰) and IAEA-602 (δ^18^O = 71.28 ‰) were used to calibrate laboratory isotope standards and to scale-normalise raw data. The laboratory standards were IAEA-CH6 cellulose (δ^18^O = 37.09 ± 0.09 ‰), commercially available cellulose powders from Merck (δ^18^O = 27.97 ± 0.08 ‰) and Fluka (δ^18^O = 28.84±0.12‰), as well as the in-house standards rice cellulose (δ^18^O = 23.64 ± 0.15 ‰) and peanut cellulose (δ^18^O = 23.93‰±0.11‰).

Isotope values are reported in δ-notation (‰) according to the equation δ = (R_S_/R_St_ - 1) where R_S_ is the isotope ratio (^13^C/^12^C, ^14^N/^15^N, ^18^O/^16^O) of the sample and R_St_ is the isotope ratio of the respective standard. δ-values are normalized to the VPDB (Vienna Pee Dee Belemnite) scale for carbon, the AIR (atmospheric nitrogen) scale for nitrogen and the VSMOW (Vienna Standard Mean Ocean Water) scale for oxygen, respectively. The overall precision of replicate analyses of the standards was <5% (rel.) for carbon, nitrogen and oxygen content, and < 0.1‰ for δ^15^N and δ^13^C and <0.25‰ for δ^18^O.

## Results

### Lithology and chronology

The lithology of core Tuz-694 is dominated by homogeneous, faintly layered peat throughout the upper three meters of the composite profile (S1 Fig). The lower part of the profile is characterized by intercalations of layers of peat and clastic material of variable thicknesses. Below a depth of 699 cm (7200 cal yr BP), the organic carbon content of the samples was very low and clastic material dominated the core. Thus, analyses were not performed beyond this depth.

**Fig 2:**
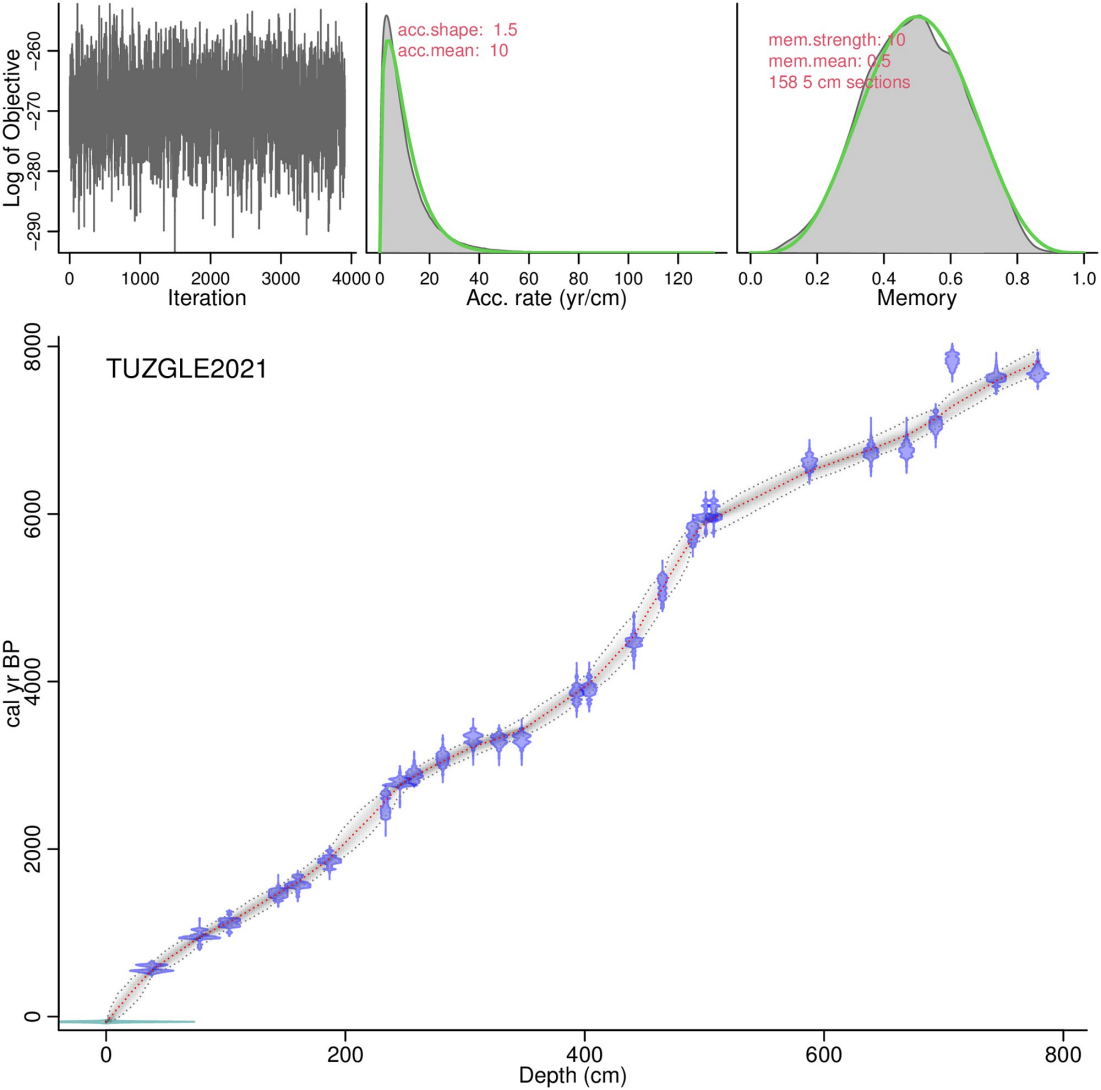
Bacon age-depth model for the CTP composite profile. Upper panels depict the **Markov Chain Monte Carlo** (MCMC) iterations (left), the prior (green curves) and posterior (grey histograms) distributions for the accumulation rate (middle panel) and memory (right panel). The bottom panel shows the calibrated ^14^C dates (light blue) and the age-depth model (red curve) with 95% confidence intervals (grey dotted lines).

The age model for the CTP composite profile (Fig 2) reveals a mean sedimentation rate of 0.11 cm/a, with variations between 0.04 cm/a and 0.22 cm/a. Maximum sedimentation rates occur between 3400 cal yr BP and 3220 cal yr BP (345 cm to 306 cm), while minimum sedimentation rates occur between 5720 cal yr BP and 4650 cal yr BP (490 cm to 446 cm). Sedimentation rates are also low from 560 cal yr BP to the present (39 cm to 0 cm) and between 2760 cal yr BP and 1970 cal yr BP (245 cm to 192 cm), while they are mainly above the mean value before 5900 cal yr BP (below 503 cm).

### Plant macrofossils

The two most common plant remains, which make up close to 100% of the peat matrix, are tube-like remains of rhizomes and roots of the Juncaceae genus *Oxychloe* and the Cyperaceae tribe Scirpeae (including *Eleocharis*, *Phylloscirpus* and *Zameioscirpus*). The current vegetation at the coring site is dominated by *Oxychloe andina* with abundances of about 80–90% [25, 33]. Drier and/or disturbed locations show higher abundances of *Zameioscirpus muticus* [34].

Throughout the entire 7200-yr peat record, *Oxychloe* was the dominating peat forming plant (S2 Fig). Higher abundances (max. 50%) of Scirpeae (presumably *Zameioscirpus*) only appear during relatively short time periods between approximately 6900–6600, 5900–5700, 4000–3800, 3400–3000, 2400, and 1400–900 cal yr BP.

### Geochemistry

**Fig 3:**
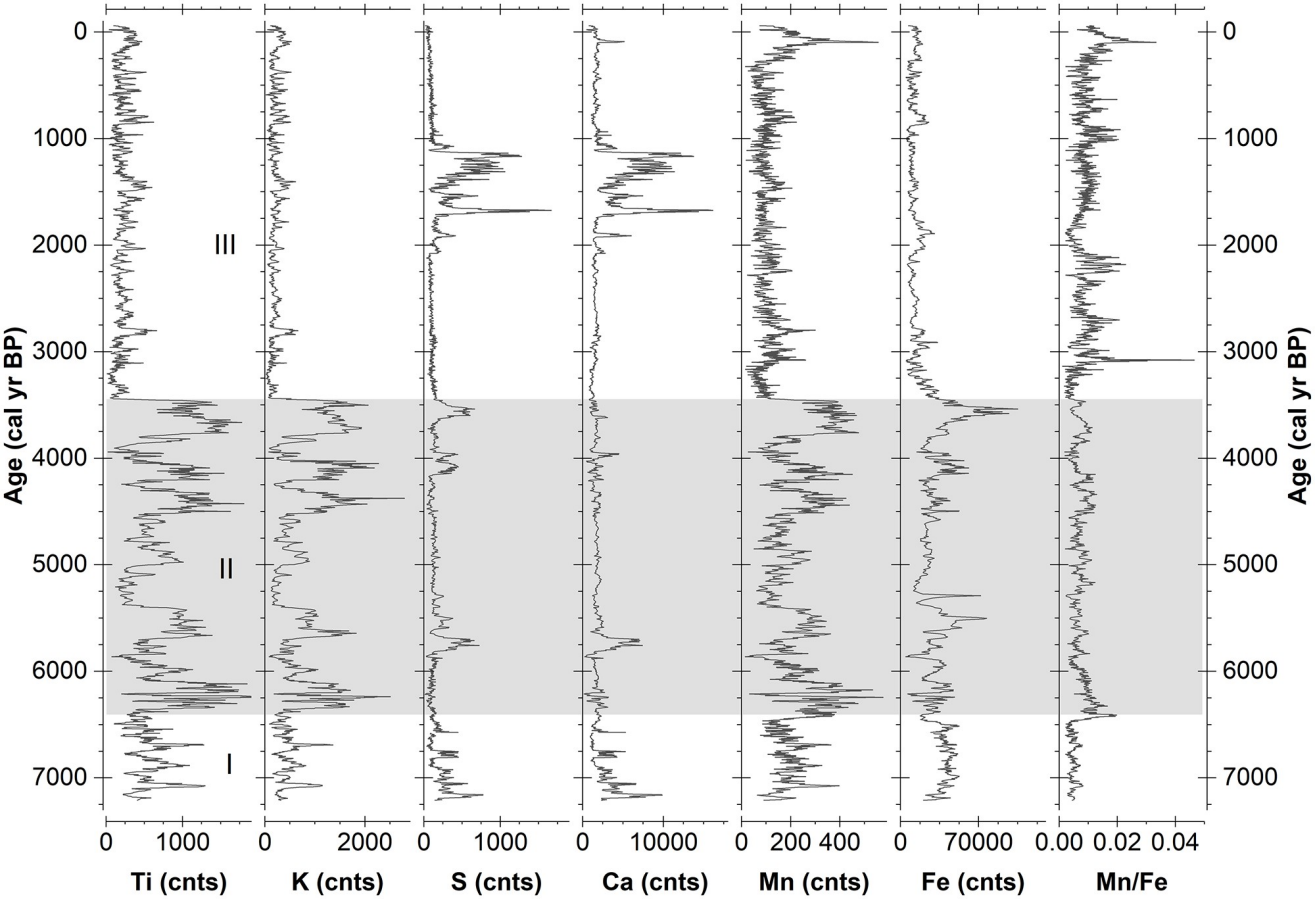
Minerogenic components of the CTP composite profile. Shown are titanium (Ti), potassium (K), sulphur (S), calcium (CA), manganese (Mn), iron (Fe) and the Mn to Fe ratio (Mn/Fe). The grey bar indicates the period with considerable fluctuations of clastic elements.

Counts of detrital minerogenic matter represented by elements such as titanium (Ti) and potassium (K) divide the profile into three consecutive segments (Fig 3). In comparison, moderate variations and intermediate element counts characterize the lowermost segment of the composite core from 7200 cal yr BP to 6390 cal yr BP. Absolute maxima and minima of the respective element counts and extreme fluctuations with several strong and distinct switches between the maximum and minimum values characterize the following section between 6390 cal yr BP and 3450 cal yr BP. This is in stark contrast to the uppermost section, which is characterized by consistently low element counts and the lowest variations observed throughout the profile.

Potentially wind-borne or evaporation-sensitive elements sulphur (S) and calcium (Ca) show somewhat elevated counts at the core bottom between 7200 cal yr BP and 6910 cal yr BP. Further up until about 3450 cal yr BP, the profile is marked by several spikes of variable but moderate magnitude. The key feature of the S and Ca records is a period of considerably rising element counts between 2080 cal yr BP and 1100 cal yr BP in the upper part of the profile (Fig 3). Kock et al. [22] interpreted this feature as a period of increased evaporation and secondary precipitation of gypsum within the cushion.

Weathering and redox-sensitive elements such as iron (Fe) and manganese (Mn) follow the partitioning determined by the detrital elements (Fig 3). This similarity is stronger for Mn counts, indicating that manganese distribution throughout the core is coupled to detrital input. An exception to this is a distinct peak in the upper part of the core 660 cal yr BP to present, which is hardly visible in the Ti and K records and not visible for iron. Fe deviates from Mn as counts in the section between 6390 cal yr BP and 3450 cal yr BP show considerably less fluctuation and a distinct maximum centred at 3540 cal yr BP. The general pattern is comparable between the two elements with a clearly expressed boundary at 3450 cal yr BP. Correspondingly, Mn/Fe ratios show low variability with distinct peaks occurring at 3080 cal yr BP and 130 cal yr BP. While the boundary at 6390 cal yr BP is expressed as a distinct rise, the poorly developed boundary at 3450 cal yr BP is highlighted by minimum values and a delayed increase. Overall, the section from 3450 cal yr BP to the present shows elevated Mn/Fe ratios compared to the other sections and several well-expressed fluctuations.

### Stable isotopes and element contents

**Fig 4:**
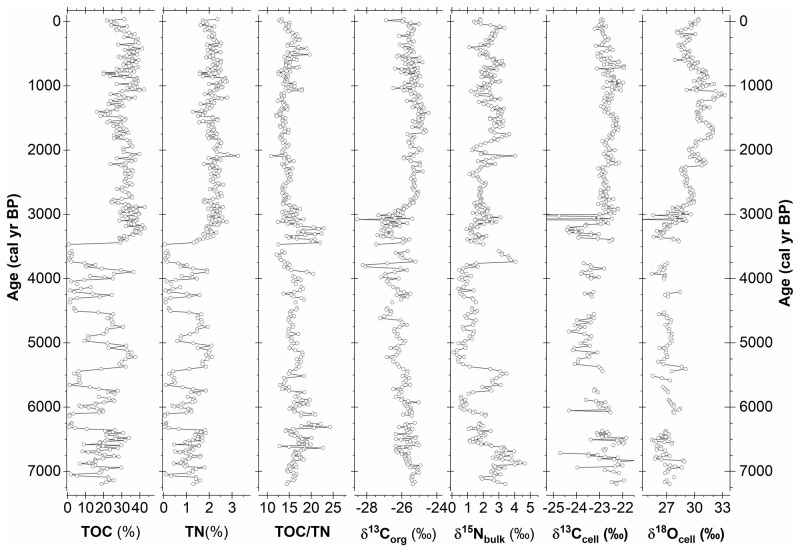
Element contents and stable isotope data of the CTP composite profile. Shown are the total organic carbon content (TOC), total nitrogen content (TN), the TOC to TN ratio (TOC/TN), the stable isotope composition of total organic carbon (δ^13^C_org_), the stable isotope composition of total nitrogen (δ^15^N_bulk_), the stable carbon isotope composition of cellulose (δ^13^C_cell_) and the stable oxygen isotope composition of cellulose (δ^18^O_cell_).

Total organic carbon content in the CTP profile varies from 0.7% to 42.8%, indicating an alternation between clastic and organic matter-dominated phases, and thus shows a high level of similarity with the Ti record (Fig 4). From 7200 cal yr BP to 6390 cal yr BP, TOC fluctuates around 20% with highest values exceeding 30% and lowest values of about 2%. Centennial-scale periods of extremely low TOC concentrations (<2.0%) mark the onset and end of the following section between 6390 cal yr BP and 3450 cal yr BP. Several distinct alternations between such low and high TOC contents (>35%) further characterize this section. Organic matter dominates the uppermost section between 3450 cal yr BP and the present. Here, the highest TOC concentrations exceed 40.0% while the lowest values barely fall below 20%. Low TOC concentrations between 1700 cal yr BP and 1300 cal yr BP coincide with massively increased Ca and S element counts, thus indicating dilution with inorganic precipitates (gypsum). The arithmetic mean TOC for the uppermost section is 32.5%, corresponding to an organic matter content of 65% in accordance with a conversion factor (1% TOC ≅ 2% OM) derived from loss on ignition (550°C) measurements (not shown). The respective TN record is an almost perfect image of TOC with the Pearson’s correlation coefficient determined as r = 0.95 (p = <0.005). The respective TOC/TN ratios show low amplitude variations around an arithmetic mean of 15.0. Visibly higher values occur during a short period with multiple peaks from 3450 cal yr BP to 3200 cal yr BP and around 6470 cal yr BP, while lowest ratios occur at 3700 cal yr BP. Overall, TOC/TN ratios are slightly elevated in the lower part of the core until 3450 cal yr BP compared to the upper part.

δ^13^C_org_ values (Fig 4) mainly vary from -27.5‰ to -24.7‰ with single extremes declining to -28.5‰ (3080 cal yr BP). From an initial level of -25.5‰, values start to decrease around 6040 cal yr BP, reaching a level of approximately -27.5‰ at 3450 cal yr BP. This decline is accompanied by an increase in variance, mainly due to decreasing relative minima, and several centennial-scale oscillations. The following significant increase lasts until 2700 cal yr BP with δ^13^C_org_ again reaching values of -25.2‰. From then on, δ^13^C_org_ values mainly remain on a level of -25.5 ±0.5‰ until the present. A smaller positive shift and temporally increased variance occur after 1700 cal yr BP.

δ^15^N_bulk_ values vary between 0.1‰ and 4.7‰. While δ^15^N_bulk_ values overall remain around 3.2‰ until 6600 cal yr BP, they decline to values around 1.6‰ thereafter (Fig 4). An extended period of the lowest values around ~1.0‰ lasts from 6040 cal yr BP to 3800 cal yr BP, followed by increasing values until about 2000 cal yr BP. Two phases with markedly elevated δ^15^N_bulk_ centred around 5510 cal yr BP and 3700 cal yr BP interrupt this period of low δ^15^N_bulk_ values. After 2000 cal yr BP, values fluctuate around 2.5‰ (±0.7‰) until the present.

δ^13^C_cell_ primarily vary from -24.5‰ to -22.0‰ with single extremes declining to -28.1‰ (3020 cal yr BP) (Fig 4). Surpassed by several mainly negative extremes, values fluctuate within a range of -23.0‰ to -21.8‰ until 6390 cal yr BP. From then on, values decline towards a level of -23.7‰ ± 0.5‰ around 4840 cal yr BP and slightly increase thereafter until 3760 cal yr BP. Between 3760 cal yr BP and 3430 cal yr BP, no cellulose data are available as the extracted cellulose yields were insufficient. At 3430 cal yr BP, a V-shaped negative excursion of nearly 2‰ starts, from -22.5‰ down to -24.4‰ and back to -22.7‰, which is again reached at 3150 cal yr BP. In either case, considerably higher δ^13^C_cell_ values than before are reached much earlier and faster compared to δ^13^C_org_ (3150 cal yr BP compared to 2700 cal yr BP). From 3430 cal yr BP to the present, δ^13^C_cell_ values show several oscillations around a mean of -22.7‰ with maximum values reached at around 1000 cal yr BP. The correlation between δ^13^C_cell_ and δ^13^C_org_ amounts to r = 0.60 (p = <0.005). The relocation of mobile degradation products such as humic acids along the profile possibly explain the differences between the two carbon isotope records and especially the slower increase of the δ^13^C_org_ values after 3430 cal. years BP.

δ^18^O_cell_ values show a considerable range of 8‰, varying mainly between 25.4‰ and 33.3‰ (Fig 4). A single extreme value even declines to 23.4‰ at 3080 cal yr BP. Until 6390 cal yr BP, δ^18^O_cell_ values show variations around a mean of 26.9‰ (25.4‰– 29.0‰). From then on, values show a slightly declining trend towards 26.7‰ until 3800 cal yr BP. Comparably weak but visible longer-term oscillations and several extreme values characterize this trend. Again, between 3760 cal yr BP and 3430 cal yr BP, no cellulose data are available due to insufficient cellulose yields. Immediately after 3430 cal yr BP, values of around 28.2‰ occur, but the main feature is a 4.0‰ increase in δ^18^O_cell_ values starting from 26.0‰ at 3350 cal yr BP and reaching 30.0‰ at 2800 cal yr BP. While the start of this increase coincides with respective increases of δ^13^C_org_ and δ^13^C_cell_, the pace of the development is intermediate between those two variables. Unique to the δ^18^O_cell_ is the higher level reached at the end of the rise compared to the period between 7200 cal yr BP and 6390 cal yr BP. After 2800 cal yr BP, the values continue to increase following a short decline until the δ^18^O_cell_ reaches a maximum at 1150 cal yr BP. The subsequent decline is similarly strong and lasts until 350 cal yr BP at which point the values start to increase again. A correlation coefficient of r = 0.49 (p = <0.005) for the δ^18^O_cell_ and the δ^13^C_cell_ does not suggest a single common controlling factor such as stomata aperture.

Overall, the pattern emerging from our data divides the CTP profile into three parts that are separated by transitions of different magnitude. While the transition starting at 3430 cal yr BP is well expressed in almost all variables by rather strong alterations, less proxies and weaker alterations characterize the transition at 6390 cal yr BP. The lowest section of the profile until 6390 cal yr BP occupies an intermediate position between the two upper sections with moderate alternations between clastic and organic matter, high stable carbon isotope values but low stable oxygen isotope values. Strong alternations between phases of clastic or organic matter dominance and overall low values of stable carbon and oxygen isotope values characterize the following section until 3430 cal yr BP. From then on until the present, organic matter completely dominates the lithology, accompanied by continuously high values of carbon and oxygen stable isotope proxies.

## Interpretation and discussion

### CTP hydroclimatic record

**Fig 5:**
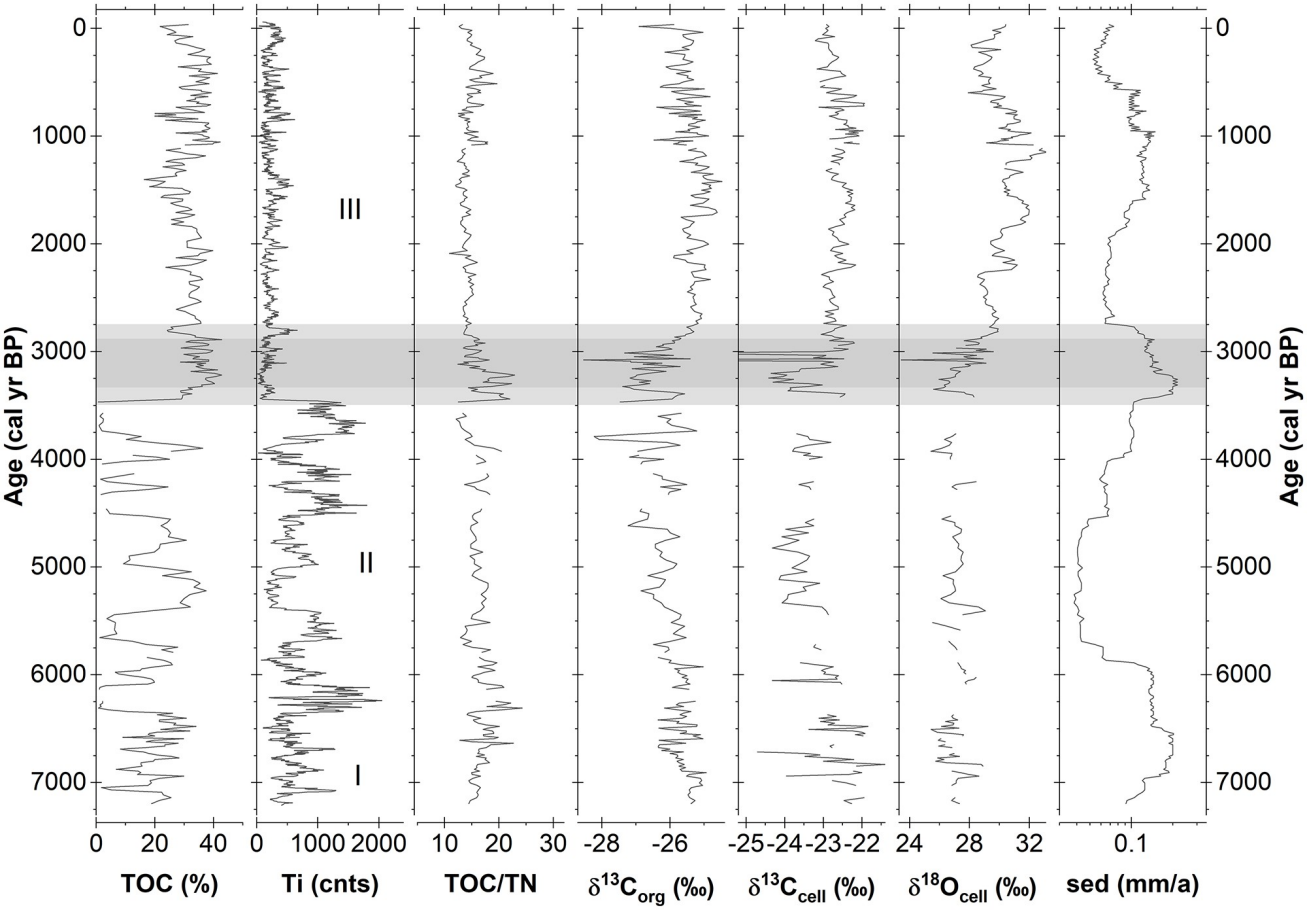
Synthesis of hydroclimatic proxies of the CTP record. Shown are the total organic carbon content (TOC), titanium (Ti), TOC/TN, the stable carbon isotopes of total organic matter (δ^13^C_org_) and cellulose (δ^13^C_cell_), the stable oxygen isotopes of cellulose (δ^18^O_cell_) and the sedimentation rate (sed, log scale). The grey bars indicate the hydroclimatic transition, as mentioned in the text.

In the semi-arid environment of the Altiplano, hydroclimate limits the capacity and the conditions of the cushion plants for growth and biomass production, which, in turn, are reflected in growth and in biogeochemical, namely isotopic, signatures of accumulated organic matter respectively peat. Organic matter contents of up to 65% indicate favourable conditions for cushion plants and continuously high accumulation of organic matter during the last 3450 cal yr BP (Fig 5). This is in contrast to the lower overall organic matter contents throughout the middle Holocene, indicating comparably less favourable conditions for plant growth. Severe aridity with prolonged periods of dormancy and interrupted organic matter production highlighted by centennial-scale phases of clastic matter dominance are particularly characteristic between 3450 cal yr BP and 6390 cal yr BP. We assume that mass movement events and torrential debris flows in conjunction with individual severe precipitation/snowmelt events are likely to have contributed to those alternating organic and clastic facies.

In parallel with the onset of the organic matter-dominated facies at 3450 cal yr BP, carbon isotope values of organic carbon and cellulose increase and reach distinctively higher values than before (Fig 5). In general, stable carbon isotope ratios of vascular plant organic matter are governed by photosynthetic carbon demand and stomata aperture and, in this respect, reflect primary production [27, 44]. Considering SASM summer precipitation supply to the CTP during the last 2900 cal yr BP [22, 23], lower TOC contents, debris flows and decreased carbon isotope values observed in the CTP before 3450 cal yr BP until 6390 cal yr BP indicate comparably decreased plant growth and, most likely, decreased mean moisture availability. Several other factors may lead to a respective change in carbon isotope composition of the deposited peat, but these can plausibly be ruled out. (i) Antarctic ice core records indicate no relevant changes for the concentration or isotopic composition of atmospheric CO_2_, i.e. the inorganic photosynthetic carbon source, for the middle Holocene [45]. (ii) Bulk peat δ^13^C was interpreted as a growing season temperature proxy with a factor of 0.97 ± 0.23‰/°C in a *Distichia* peatland of the Peruvian Andes [26]. This would transfer into a comparatively high ~2°C increase in growing season temperature from the middle to late Holocene at the CTP. To our knowledge, no other studies indicate such strong temperature changes for the region. Nevertheless, a prolongation of the growing season enabled by an improved water supply during summer months would induce a similar “temperature” effect. (iii) Sustained changes in peat-forming vegetation may induce changes in the δ^13^C signature of deposited organic matter [22, 46], but such vegetation changes are not indicated by the macrofossil record of the CTP (S2 Fig). (iv) Increasing (decreasing) carbon isotope values of vascular plant organic matter may also indicate increased (reduced) water stress or aridity. Decreasing (increasing) water availability would lead to reduced (increased) stomata aperture, reduced (increased) gas exchange between the leaf internal pore space and the atmosphere, a reduced (increased) leaf internal carbon dioxide concentration and, therefore, less (increased) fractionation against ^13^C [44, 47]. However, rising δ^13^C values together with an assumed increase of plant growth, as observed for the CTP, contradict such a hypothesis. Furthermore, growth chamber experiments revealed reduced ambient photosynthetic rates and slightly decreased δ^13^C_cell_ values under drought conditions for Eucalypt seedlings [48]. (v) The plant organic matter carbon isotope signal that is consistently preserved after early diagenesis may be biased by degradation products such as humic acids that are more depleted compared to their precursor materials [49] if those materials are preferably deposited during certain time frames. Since a disproportionate admixture of degradation products would only alter the δ^13^C_org_ signal, such an effect is ruled out by the broadly constant offset between δ^13^C_org_ and δ^13^C_cell_ (Δ^13^C_cell-bulk_: 2.0‰ to 4.0‰, not shown) observed for the CTP.

The longer term δ^18^O composition of vascular plant organic matter is primarily affected by the evaporative enrichment of leaf water and source water δ^18^O variations [50]. Irrespective of the potential degree of leaf water isotope enrichment, an observed leaf water enrichment of 4.5‰, induced by drought stress and low humidity conditions, led to a respective response of only 0.6‰ in the δ^18^O of stem cellulose in a growth chamber experiment with Eucalypt seedlings [48]. Furthermore, a study on *Larix decidua* in Switzerland revealed source water δ^18^O to be the dominant factor for seasonal variations in tree-ring δ^18^O, while short-term variations in needle-water δ^18^O enrichment were not reflected in the tree-ring [51]. In view of these results, strong variations of 2.0‰ to 4.0‰, as observed in δ^18^O_cell_ of the CTP profile around 3100 cal yr BP, can hardly be explained by assuming leaf water isotope enrichment as the leading driver of variance.

Biochemical fractionation during photosynthesis and re-equilibration during cellulose synthesis enriches the oxygen isotope signature of plant cellulose against the respective source water by about 27‰ [52]. Several field studies have shown that δ^18^O values of vascular plant organic matter in peat environments offer a fairly good reflection of the isotopic composition of precipitation and peat water [27, 53, 54]. Larger changes (beyond seasonal or longer-term natural variations) in source water isotopic composition for cushion peatland plants may arise from alterations in local surface water evaporation or from changes in the moisture (precipitation) source region. We estimate that the influence of enriched surface water on cushion plant metabolic water and δ^18^O_cell_ is limited to a positive shift of about 1.5‰ [22, 27]. This view is supported by the fact that lateral hydrologic connectivity near the peatland surface seems limited, thus explaining large differences in the open water isotopic composition observed for the CTP. Centennial-scale temporal variations of the degree of surface water evaporation are also likely to be small as long as semi-arid conditions prevail. Specifically, the increase of more than 6.0‰ in the δ^18^O_cell_ observed for the CTP between 3350 cal yr BP and 1200 cal yr BP is hardly plausible explained by changes in surface water evaporation alone (Fig 5).

This suggests that a change of the dominant moisture source is the most plausible hypothesis to explain the observed δ^18^O_cell_ shift starting around 3350 cal yr BP. What evidence supports this hypothesis? During the last 2900 years, the CTP has obtained moisture mainly derived from the SASM [22, 23]. Convective activity and rainout over the Amazon basin together with further upstream processes, especially partial rainout and vapour recycling, were primary controls on the isotopic composition of SASM precipitation in the central Andes [55–58]. Overall, positive precipitation anomalies coincide with comparably negative δ^18^O_precip_ anomalies on the southern Altiplano even if the relation between precipitation amount and δ^18^O_precip_ locally varies [56, 57]. Significant negative correlations also appear on multi-decadal timescales between tree-ring δ^18^O chronologies from *Polylepis tarapacana* and precipitation amount in the Chilean and Bolivian Altiplano [59]. As the amount of precipitation is negatively correlated with δ^18^O_precip_, more negative (positive) δ^18^O values indicate increased (decreased) amounts of SASM precipitation [56, 60, 61]. Accordingly, the δ^18^O_cell_ variations observed at CTP during the last ~2800 cal yr BP reflect SASM derived moisture variability presumably with a grossly invariant moisture source area in the Amazon basin (tropical Atlantic). This mechanism cannot explain the strong decline in δ^18^O_cell_ values (~4‰) observed from 2800 cal yr BP to 3430 cal yr BP, as this would imply much stronger SASM activity and higher precipitation amounts before 3430 cal yr BP than after 2800 cal yr BP.

We therefore interpret this change in δ^18^O_cell_ as a transition between different prevailing modes of atmospheric circulation, determining the precipitation/moisture regime of the CTP respectively. Before 3430 cal yr BP, a mode delivering comparably less precipitation from isotopically depleted moisture sources was predominant, while thereafter the SAMS delivered more and comparably isotopically enriched precipitation reaching its full strength around 2800 cal yr BP. We can assume two potential moisture sources for depleted precipitation at the CTP during the middle Holocene before 3430 cal yr BP. Moisture crossing the Andes from westerly directions is strongly affected by orographic uplift and progressive rainout. Consequently, precipitation in the Andes and the Eastern Cordilleras originating from this moisture can be isotopically heavily depleted [62, 63]. Thus, moisture originating in the Pacific and reaching the CTP from westerly directions with the SHW could be a possible source of depleted precipitation under circulation conditions of the middle Holocene. This potential pathway is exemplified by the fact that at present, SHW winter precipitation east of the Andes (e.g. 30°S) is isotopically more depleted than the respective SASM summer precipitation further north (e.g. 24°S) in an order of 2–6‰ [61, 64]. An alternative source for isotopically light precipitation could potentially be moisture originating from the subtropical or subpolar Atlantic, influenced by the entrainment of recycled moisture during transport [55, 65] reaching the CTP from SE directions. The available evidence for such a path is admittedly limited and the potential degree of depletion through recycled moisture needs further verification. The slightly declining trend in δ^18^O_cell_ towards 3430 cal yr BP could indicate a strengthening impact of precipitation from westerly directions within this context. Irrespective of its general absence, temporally increased δ^18^O_cell_ values between 3430 cal yr BP and 6400 cal yr BP could well be explained by a contribution of SASM-derived precipitation that sporadically reached the CTP.

We therefore suggest that CTP received depleted moisture either from the SW (SHW) or from the SE (recycled moisture, subtropical Atlantic) until 3430 cal yr BP. During that period, moisture from the SASM was scarce but might be the cause for shorter periods with somewhat increased δ^18^O_cell_ values. Conditions were dry and growth of cushion plants was limited with a comparably low photosynthetic carbon demand. Vegetation periods were most likely shortened due to summer and autumn dryness. The transition towards an SASM-dominated summer precipitation regime at the CTP started around 3430 cal yr BP, as indicated by the continuous increase in δ^18^O_cell_ values. The development was likely smoothed by a mixing effect between the two precipitation endmembers (“SHW”, SASM) in the shallow groundwater reservoir feeding the peatland. The transition was completed around 2800 cal yr BP. Since 2800 cal yr BP, the SASM has consistently reached the CTP, bringing more and enriched moisture from northerly directions but semi-arid conditions still prevail. An improved water supply fostered plant growth, leading to peat growth and a vertical and horizontal expansion of the peatland. Photosynthetic carbon demand was therefore considerably increased, whereas still effective water limitation continued to strongly constrain compensating increases of stomata aperture. As a consequence of enhanced peat growth, debris flows from strong precipitation events no longer reach the coring position on the peatland.

### Regional comparisons

**Fig 6:**
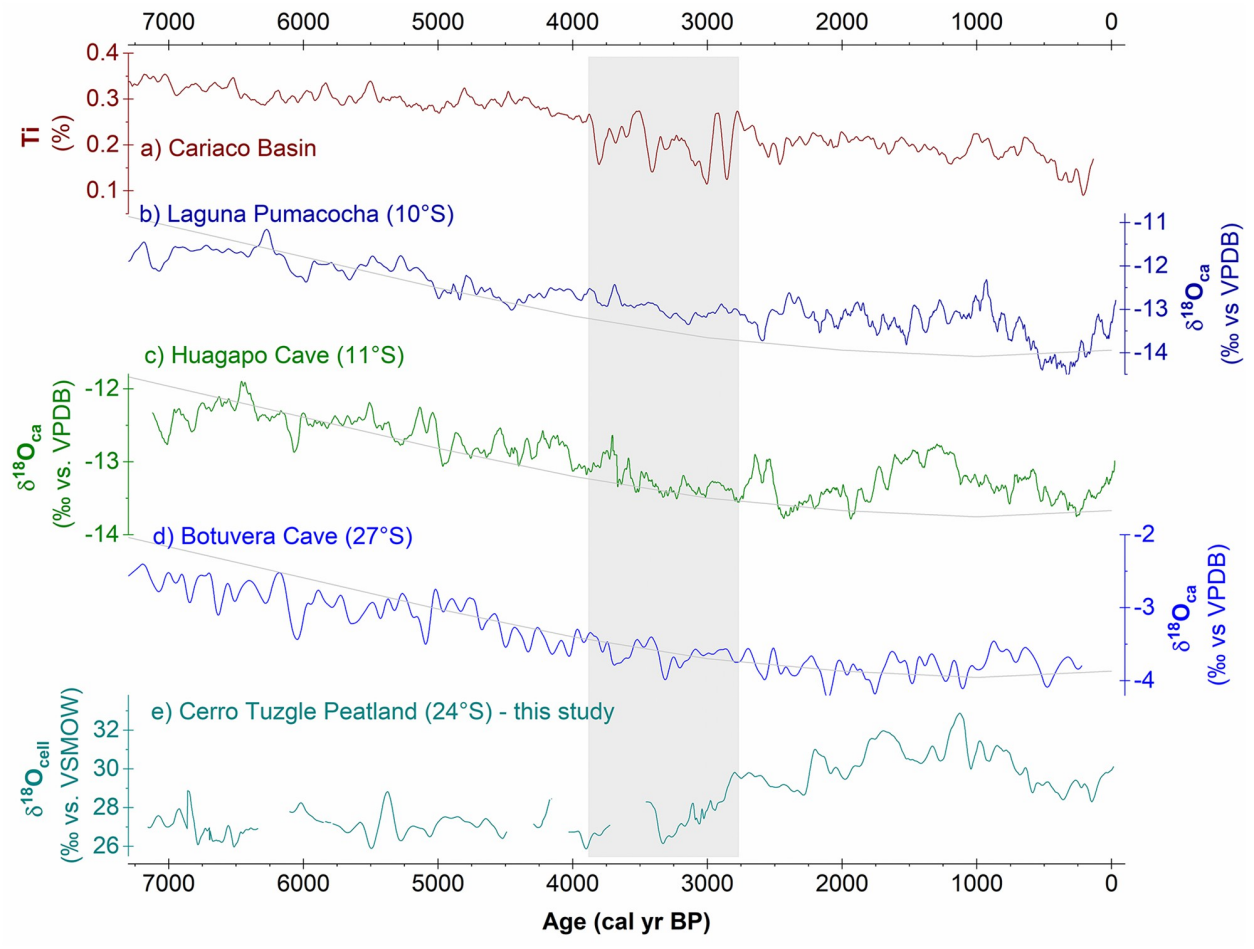
CTP record compared to proxy records for the ITCZ position and the SASM. **(a)** Cariaco Basin Ti content as proxy for the ITCZ position [71]. **(b)** Lacustrine carbonate δ^18^O of Laguna Pumacocha, Peru [67, 68], **(c)** stalagmite δ^18^O from Huagapo cave, Peru [69], and **(d)** stalagmite δ^18^O from Botuvera cave, SE Brazil [66] as proxies for SASM strength (lower values indicate intensified SASM). December insolation at 30°S [72] indicated on reversed scale as grey line (scaling from 465 to 490 W/m^2^). **(e)** Cellulose δ^18^O from CTP, Argentina, as hydroclimatic proxy. The grey bar indicates the window of transition mentioned in the text. All records are annualized and averaged with a 51-year moving average window.

Valuable information on systematic differences and similarities of the South American Holocene hydroclimate can be obtained by contrasting the development of the CTP with SASM records further north and east and with the position of the ITCZ (Fig 6). Stalagmite stable oxygen isotope (δ^18^O_ca_) records from Huaguapo and Botuvera cave and lacustrine carbonates from Laguna Pumacocha [66–69] represent regions outside of the central Amazon Basin where the oxygen isotope composition is dominated by the SASM. For much parts of the Holocene, the stalagmite δ^18^O records share a common decreasing trend that explains about 50% of common variance and corresponds to increasing austral summer insolation [70]. The continuous decrease in δ^18^O values (Fig 6) was interpreted as an increase in the SASM strength that is mainly driven by the Holocene increase in austral summer insolation and the position of the ITCZ [66, 69]. The continuous decline came to a halt around 3000 cal yr BP [70] and δ^18^O_ca_ values thereafter broadly remained low with several fluctuations. A similar development is revealed by lacustrine carbonate δ^18^O_ca_ values from Laguna Pumacocha (Fig 6). The increase in austral summer insolation also slowed down around 3000 cal yr BP and reached a maximum around 2000 cal yr BP [67, 68]. The prominent transition in the δ^18^O_cell_ record of the CTP is centred around 3100 cal yr BP and occurs exactly within the time frame where stalagmite δ^18^O_ca_ records and austral summer insolation reach a plateau (Fig 6). Meanwhile, δ^18^O_cell_ values of the CTP do not show a trend comparable to stalagmite δ^18^O_ca_ records before 3430 cal yr BP. This is consistent with the notion of an increase in SASM strength and extension during the Holocene only reaching the latitudes of the CTP around 3430 cal yr BP. Before that time, the CTP was therefore outside of the SASM sphere of influence. During a transition period, the SASM regime developed consistently and became fully established at the CTP around 2800 cal yr BP. Situated at its current southern limit, the CTP does accordingly not reflect the Holocene strengthening of the SASM, but instead shows a threshold response to SASM forcing being either in or outside of the SASM realm.

In line with austral summer insolation, an increasingly southerly position of the ITCZ throughout the Holocene is indicated by the Cariaco Ti record [71]. While the Ti record shows a rather smooth development overall, repeated high-amplitude fluctuations stand out in the period 3800 cal yr BP to 2800 cal yr BP. A noticeable match emerges in comparison to CTP with respect to the temporal setting of the transition period (3430 cal yr BP to 2800 cal yr BP) as well as to high amplitude variations occurring during the transition, which are especially visible in the CTP δ^13^C_org_ record (Figs 5 and 6). The coincidence of periods of major disturbance over a large latitudinal distance appears to indicate reorganisations of major components of Southern Hemisphere atmospheric circulation and climate. This further supports our assumption of a hydroclimatic switch towards SASM summer precipitation at the CTP within this period.

Large-scale meridional atmospheric overturning over South America is realized through the South American Hadley circulation and the extratropical Ferrel circulation, inevitably linking tropical circulation in the form of SASM to extratropical circulation, namely SHW. Accordingly, changes in the South American Hadley circulation and the associated SASM could potentially cause a response in the latitudinal position or extension of the SASDZ (arid diagonal). If that is the case, the major transition observed at CTP should also be visible in archives documenting the variability of the SHW south of this zone.

**Fig 7:**
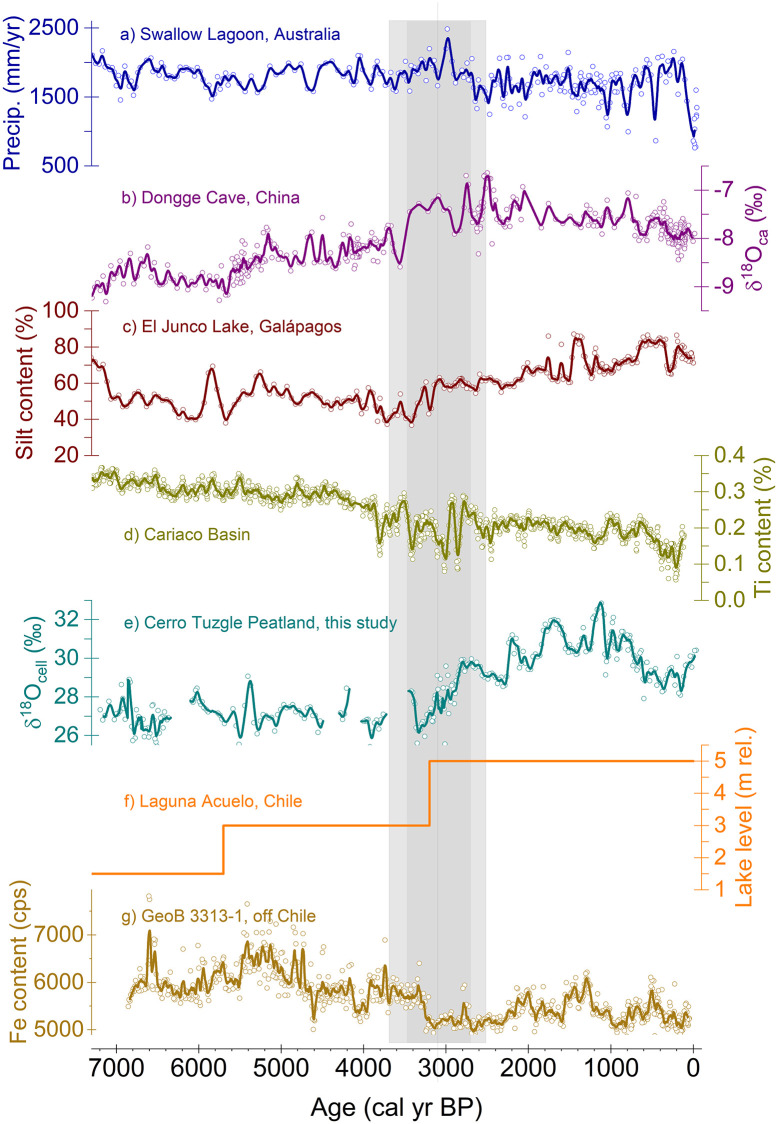
CTP record and Circum Pacific archives. **(a)** Subtropical Australian precipitation as proxy for Holocene ENSO behaviour [80]. **(b)** Stalagmite δ^18^O from Dongge Cave, southern China, as proxy for strength of Asian monsoon [79]–increasing values indicate decreasing precipitation. **(c)** Silt content of El Junco lake sediment, Galápagos Islands [78] as proxy for Holocene ENSO behavior–increasing silt content indicates increasing precipitation. **(d)** Cariaco Basin Ti content as proxy for the ITCZ position [71]. **(e)** Cellulose δ^18^O from the CTP, Argentina, as hydroclimatic proxy. **(f)** Lake level of Laguna Acuelo, central Chile [73]–higher lake level indicates increasing precipitation. **(g)** Iron content of marine core GeoB 3313–1 off Chile as proxy for rainfall variability derived from SHW [14]–lower iron contents indicate more humid conditions. Note that the age model of GeoB 3313–1 was recalculated with Bacon based on the marine radiocarbon age calibration curve Marine20 [75]. The records are annualized and averaged with a 51-year moving average window (thick coloured lines) where applicable. The grey bar indicates the window of transition discussed in the text.

Laguna Acuelo (33.5°S) is positioned south of the SASDZ in the Mediterranean zone of Chile, near the present-day northern limit of the SHW. Lake-level reconstructions of Laguna Acuelo indicate that the major Holocene lake-level increase occurred around 3200 cal yr BP (Fig 7) and was accompanied by a strong decrease in halophytes [73, 74]. Villa-Martínez et al. [74] interpreted this change towards more humid conditions and increased rainfall variability around 3200 cal yr BP as the transition towards the modern Mediterranean climate in central Chile. Similar evidence also comes from a marine sediment core (GeoB 3313–1) from the Chilean continental slope further south at 41°S (Fig 7). Lamy et al. [14] interpreted the iron content of the sediments as proxy for rainfall variability in southern Chile. Around 3250 cal yr BP, a major decrease of the iron content indicates a transition towards more humid conditions and a potential equatorward shift of the SHW [14] (Age-model recalculated based on the marine radiocarbon age calibration curve Marine20 from [75] (S3 Fig), transition age in [14] is 4000 cal yr BP).

Thus, we find evidence for contemporaneous changes in moisture supply respectively precipitation north and south of the SASDZ. To rule out the possibility of mere coincidence a common mechanism is required to explain a contemporaneous strengthening of the SHW and the SASM, as precipitation amounts appear to increase in both regions (central Chile, NW Argentina). A mere latitudinal displacement of established meridional circulation cells seems insufficient to explain such a response, as it should force disparate responses north and south of the SASDZ. We hypothesize that there is a connection with the South American Hadley circulation, i.e. its strength and the position of its southern edge, and the strength and seasonal position of the South Pacific anticyclone. Nguyen et al. [18] showed that a strong relation exists between the location of the poleward sinking edge of the South American Hadley cell and ENSO during austral summer. The contraction and expansion of the South American Hadley cell is linked to the strength of the Walker circulation. La Niña-like conditions and intensified Walker circulation promote the contraction of the South American Hadley cell and drive convergence in the subtropical dry regions of the South American sector with reduced rainfall e.g. in the coastal areas of Ecuador and Peru [18]. Furthermore, both the strength and width of the South American Hadley cell and the strength of the southern subtropical anticyclones are strongly connected to sea surface temperatures of the subtropical Pacific and Atlantic [76]. In the Central Andes, enhanced SST gradients during La Niña years lead to more frequent rainfall episodes through the strengthening and southward displacement of the Bolivian High and a subdued zonal upper level westerly circulation [77].

Finally, we recognize that several archives representing climate development in the larger South Pacific region reveal a bipolar development of precipitation regimes in the western and eastern South Pacific realm around 3100 cal yr BP (Fig 7) that could suggest potential connections with the South American Hadley cell and the Walker circulation. A clear increase in the silt fraction of El Junco sediments indicate a lasting increase in precipitation on the Galápagos Islands starting at 3200 ± 160 cal yr BP [78]. Conroy et al. [78] connected the precipitation increase to the higher ENSO frequency and longer, stronger El Niño events and, therefore, to a longer-term shift towards an ENSO El Niño state. Slightly earlier around 3550 ± 59 cal yr BP, a decline in precipitation in southern China is indicated by abruptly increasing stalagmite δ^18^O_ca_ values from the Dongge cave (Fig 7) that was attributed to a respective weakening in the intensity of the Asian monsoon [79]. Simultaneous with increasing precipitation on the Galápagos Islands, a shift towards drier climates after 3200 cal yr BP is indicated for subtropical eastern Australia (Fig 7) from a precipitation record based on carbon isotopes of a single leaf species preserved in lake sediments [80]. This shift was seen to be connected to the increasing frequency and strength of the El Niño state of the ENSO [80].

## Summary and conclusions

We investigated a cushion peatland archive located on the Argentine Altiplano at 24°S north of the present-day SASDZ spanning the last 7200 cal yr BP. Our extended CTP record revealed a remarkable transition in δ^18^O_cell_ centred at 3100 cal yr BP towards consistently increased values until the present. We attributed the increase in δ^18^O_cell_ values to a hydrological transition accompanied by changes in the source water isotopic composition. This interpretation is supported by concomitant and persistent increases of δ^13^C_org_, δ^13^C_cell_ and TOC values. Whereas the CTP region likely received depleted moisture either from the SHW within a winter precipitation regime or from the subtropical Atlantic before, the transition most likely marks the time when the SASM consistently reached those latitudes (24°S), supplying the CTP with considerably more and enriched precipitation in a summer precipitation regime.

We showed that the increase in precipitation at the CTP around 3100 cal yr BP was accompanied by increased precipitation in Chilean regions south of the SASDZ (33.5°S, Laguna Acuelo and 41°S, GeoB 3313–1) located in the winter precipitation regime of the SHW. This simultaneous alteration in tropical precipitation (SASM) and extratropical precipitation (SHW) point towards a major reorganisation of the large-scale atmospheric circulation over South America at this time. However, a mere latitudinal shift in circulation systems would not be sufficient to explain simultaneous increases in precipitation on the Argentine Altiplano at 24°S and in central Chile at 33.5°S. Further studies are needed to verify the existence of the proposed link and to explore the underlying processes and the respective forcing factors.

We also recognize that the hydrological transition at the CTP seems to be connected with changes in the larger South Pacific region. Increasing precipitation at the CTP around 3100 cal yr BP coincides with ENSO-driven increases in precipitation on the Galapágos Islands, but with decreasing precipitation in southern inland China indicating a weakening of the Asian monsoon and decreasing precipitation in subtropical eastern Australia related to the El Niño state of the ENSO.

The CTP record provides novel evidence for a major hydroclimatic transition at a latitude of 24°S around 3100 cal yr BP potentially connected to larger-scale reorganisations of South American hydroclimate and beyond. Further studies should focus on the temporal development of the described hydroclimatic transition along a latitudinal moisture gradient on the southern Altiplano and respective potential moisture sources during the middle and late Holocene.

## Supporting information

S1 Fig Stratigraphy of the composite profile of core Tuz-694 from CTP. Left panel presents the stratigraphical description of the deposited peat sediment down to 800 cm depth. The depths of the samples selected from the composite profile for radiocarbon dating are indicated (red circles) together with their respective radiocarbon ages (in red with radiocarbon error). Right panel shows the calculated age-depth model for comparison. Single core segments were carefully inspected in the field for signs of incomplete core recovery. Percussion coring causes partial compaction of peat during coring and a correction factor was applied to reconstruct the actual length of each core segment (considering only the coring material that was assessed as reliable based on visual inspection on the split cores and XRF data). To counter any effect of incomplete core recovery, compaction or caving material on the chronology, we carefully selected samples for radiocarbon dating from the top and bottom of the approved core segments. We did not observe spurious suspicious age offsets between the lower part of a respective above core segment to the upper part of a respective lower core segment. The resulting chronology consisting on 27 radiocarbon dates reveals no signs of age reversals or other potential coring problems.
(TIF)

S2 Fig Estimated proportional abundances of macrofossils of the Juncaceae genus *Oxychloe* and the *Cyperaceae* tribe *Scirpeae* (including *Eleocharis*, *Phylloscirpus* and *Zameioscirpus*) of the CTP composite profile. Abundance classes (Ab. class) visualize the relative dominance of *Oxychloe* macrofossils (>90%, >70%, >50%) along the CTP composite profile.
(TIF)

S3 Fig Bacon age model for core GeoB 3313–1 from [14]. Upper panels depict the MCMC iterations (left), the prior (green curves) and posterior (grey histograms) distributions for the accumulation rate (middle panel) and memory (right panel). The bottom panel shows the calibrated ^14^C dates (light blue) and the age-depth model (red curve) with 95% confidence intervals (grey dotted lines). The original age-depth model of GeoB 3313–1 [14] was recalculated based on the new marine radiocarbon age calibration curve Marine20 [75] to account for updates in the marine reservoir age since 2001. Bacon was used with the default settings, with the exception that the section thickness was set to 5. This set-up achieved good runs with stationary performance of iterations and reduced confidence ranges (mean 95% = ~471 yr).
(TIF)

S1 Dataset Element content and stable isotope data of CTP. Composite depth (cm), age (cal yr BP), organic carbon content (TOC), total nitrogen content (TN), TOC/TN ratio, stable carbon isotope composition of organic matter (δ^13^C_org_), stable nitrogen isotope composition of total matter (δ^15^N_bulk_), stable carbon isotope composition of cellulose (δ^13^C_cell_) and stable oxygen isotope composition of cellulose (δ^18^O_cell_). Data provided in S1 Dataset.
(XLSX)
