# Supplementary figures and images for: Hydroclimatic record from an Altiplano cushion peatland (24°S) indicates large-scale reorganisation of atmospheric circulation for the late Holocene
Source: PLoS One. 2022 Nov 10;17(11):e0277027. doi: 10.1371/journal.pone.0277027 (PMC9648708; doi:10.1371/journal.pone.0277027)

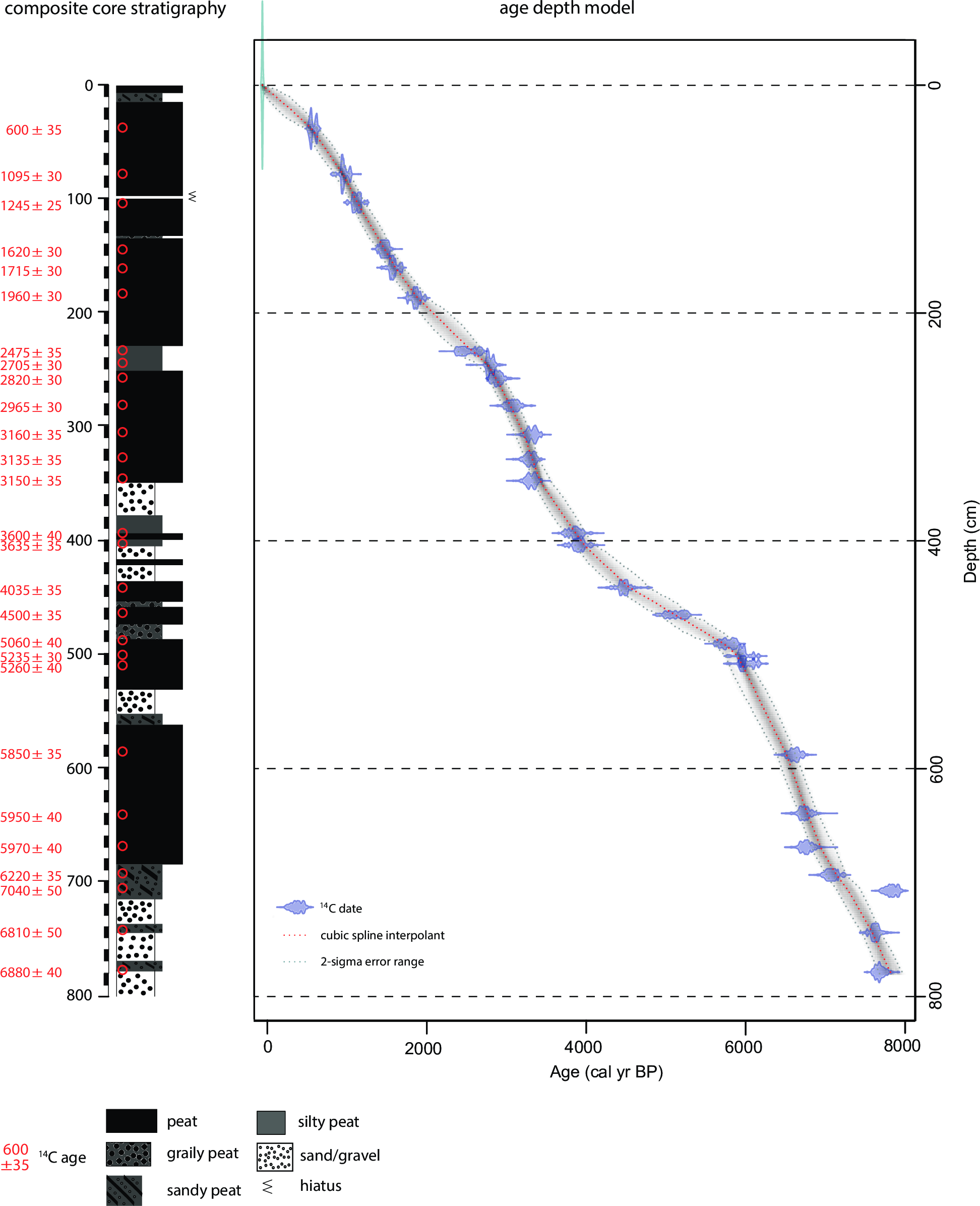

Supplement: S1 Fig — Left panel presents the stratigraphical description of the deposited peat sediment down to 800 cm depth. The depths of the samples selected from the composite profile for radiocarbon dating are indicated (red circles) together with their respective radiocarbon ages (in red with radiocarbon error). Right panel shows the calculated age-depth model for comparison. Single core segments were carefully inspected in the field for signs of incomplete core recovery. Percussion coring causes partial compaction of peat during coring and a correction factor was applied to reconstruct the actual length of each core segment (considering only the coring material that was assessed as reliable based on visual inspection on the split cores and XRF data). To counter any effect of incomplete core recovery, compaction or caving material on the chronology, we carefully selected samples for radiocarbon dating from the top and bottom of the approved core segments. We did not observe spurious suspicious age offsets between the lower part of a respective above core segment to the upper part of a respective lower core segment. The resulting chronology consisting on 27 radiocarbon dates reveals no signs of age reversals or other potential coring problems. (TIF) [file pone.0277027.s001.tif]

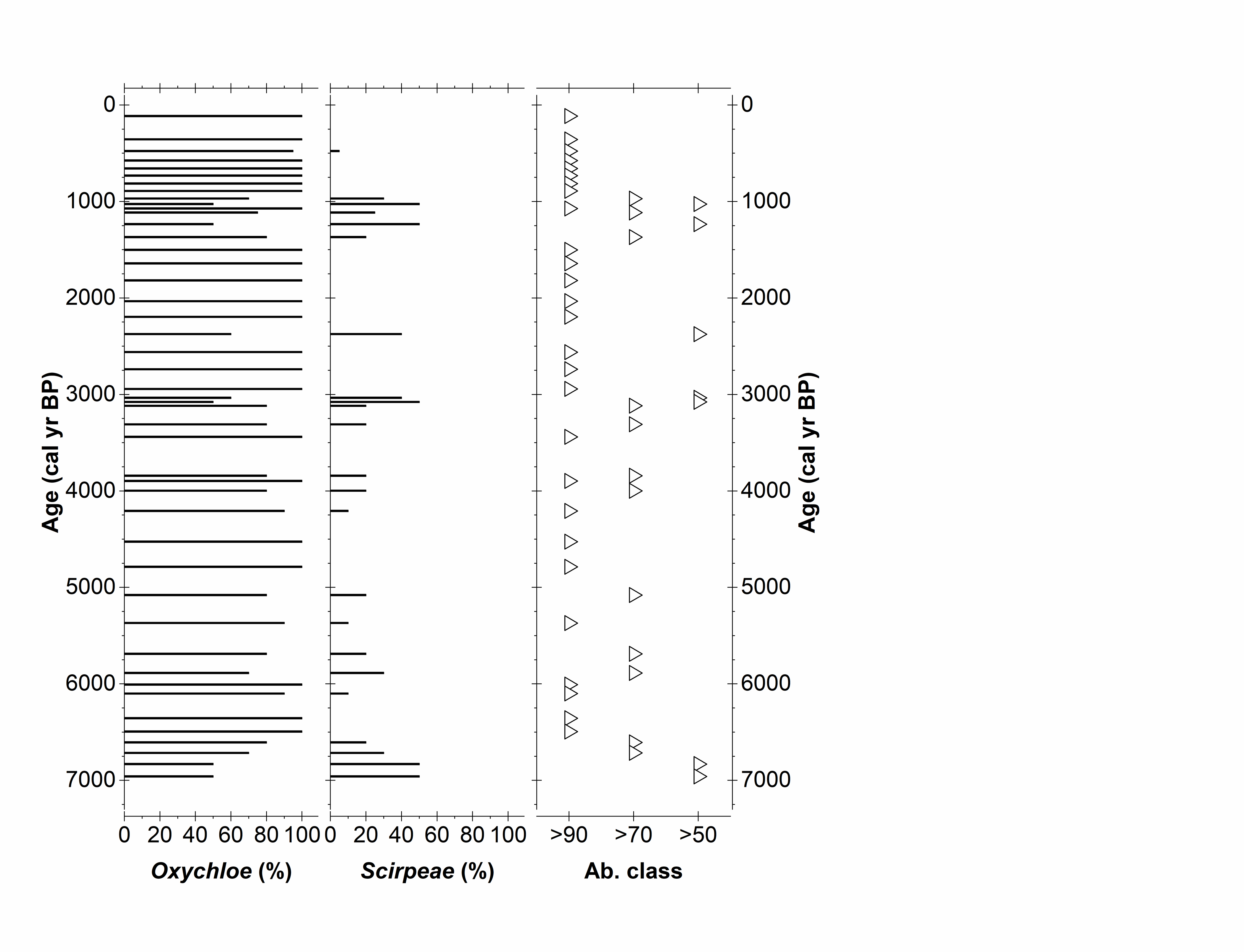

Supplement: S2 Fig — Abundance classes (Ab. class) visualize the relative dominance of Oxychloe macrofossils (>90%, >70%, >50%) along the CTP composite profile. (TIF) [file pone.0277027.s002.tif]

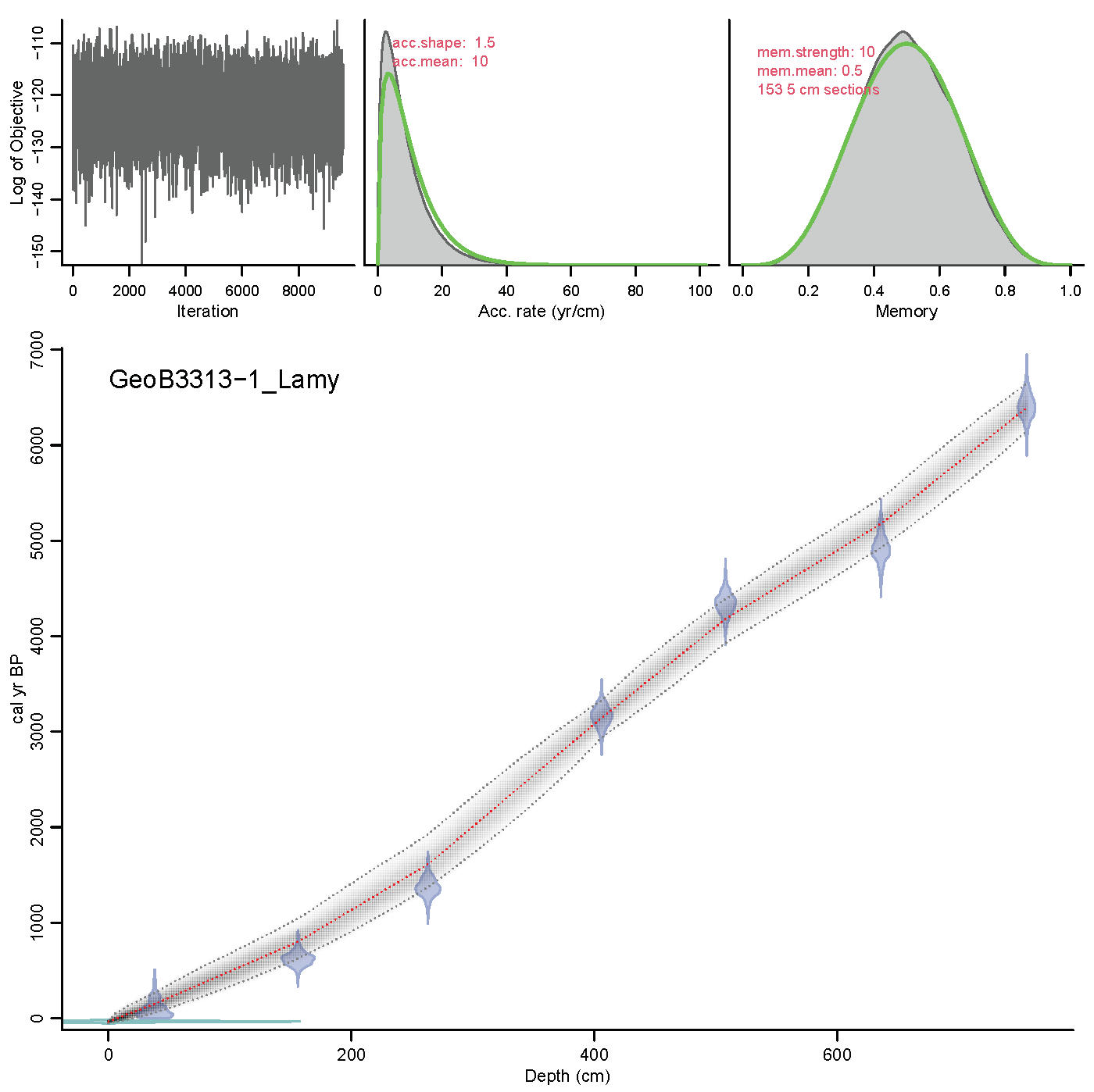

Supplement: S3 Fig — Upper panels depict the MCMC iterations (left), the prior (green curves) and posterior (grey histograms) distributions for the accumulation rate (middle panel) and memory (right panel). The bottom panel shows the calibrated 14C dates (light blue) and the age-depth model (red curve) with 95% confidence intervals (grey dotted lines). The original age-depth model of GeoB 3313–1 [14] was recalculated based on the new marine radiocarbon age calibration curve Marine20 [75] to account for updates in the marine reservoir age since 2001. Bacon was used with the default settings, with the exception that the section thickness was set to 5. This set-up achieved good runs with stationary performance of iterations and reduced confidence ranges (mean 95% = ~471 yr). (TIF) [file pone.0277027.s003.tif]
